# The language profile of formal thought disorder

**DOI:** 10.1038/s41537-018-0061-9

**Published:** 2018-09-19

**Authors:** Derya Çokal, Gabriel Sevilla, William Stephen Jones, Vitor Zimmerer, Felicity Deamer, Maggie Douglas, Helen Spencer, Douglas Turkington, Nicol Ferrier, Rosemary Varley, Stuart Watson, Wolfram Hinzen

**Affiliations:** 1 Institute of Neuroscience, Newcastle University, Newcastle upon Tyne, UK; 2 Department of Philosophy, Durham University, Durham, UK; 3 Department of Translation and Language Sciences, Universitat Pompeu Fabra, Barcelona, Spain; 4 Department of Language and Cognition, University College London, London, UK; 5 Northumberland Tyne and Wear NHS Foundation Trust, Newcastle, UK; 6 ICREA (Institució Catalana de Recerca i Estudis Avançats), Barcelona, Spain; 7 FIDMAG Germanes Hospitalaries Research Foundation, Benito Menni Hospital, Barcelona, Spain

## Abstract

Formal thought disorder (FTD) is clinically manifested as disorganized speech, but there have been only few investigations of its linguistic properties. We examined how disturbance of thought may relate to the referential function of language as expressed in the use of noun phrases (NPs) and the complexity of sentence structures. We used a comic strip description task to elicit language samples from 30 participants with schizophrenia (SZ), 15 with moderate or severe FTD (SZ + FTD), and 15 minimal or no FTD (SZ−FTD), as well as 15 first-degree relatives of people with SZ (FDRs) and 15 neurotypical controls (NC). We predicted that anomalies in the normal referential use of NPs, sub-divided into definite and indefinite NPs, would identify FTD; and also that FTD would also be linked to reduced linguistic complexity as specifically measured by the number of embedded clauses and of grammatical dependents. Participants with SZ + FTD produced more referential anomalies than NC and produced the fewest definite NPs, while FDRs produced the most and thus also differed from NC. When referential anomalies were classed according to the NP type in which they occurred, the SZ + FTD group produced more anomalies in definite NPs than NC. Syntactic errors did not distinguish groups, but the SZ + FTD group exhibited significantly less syntactic complexity than non-SZ groups. Exploratory regression analyses suggested that production of definite NPs distinguished the two SZ groups. These results demonstrate that FTD can be identified in specific grammatical patterns which provide new targets for detection, intervention, and neurobiological studies.

## Introduction

Formal thought disorder (FTD) is clinically manifest primarily as speech that is disorganized and hard to follow, exhibiting loose associations, derailment, tangentiality, or incoherence. This invites identification of precise linguistic markers of this syndrome, which would correspond to its clinical descriptors, with consideration of how language markers would relate to well-established neurocognitive impairments seen in tests of executive functioning or semantic processing.^1–4^ Previous linguistic studies of spontaneous speech in schizophrenia (SZ)^5–9^ have typically documented a reduction of syntactic complexity and increase of syntactic errors. The few studies that have compared speech in patients with and without FTD have mostly involved small samples (typically less than 11 participants with FTD) and pointed to a disorder at a semantic level of linguistic organization,^2^ involving the referential function of language in particular.^10,11^ Yet both ‘syntax’ and ‘semantics’ are broad and multi-faceted linguistic domains. Employing more specific linguistic measures of FTD, we here aimed to identify finer-grained anomalies that might enable closer integration with the neurocognitive correlates of FTD. Existing functional neuroimaging studies have provided evidence for atypical activations of regions associated with language and speech in FTD; however, functional tasks have rarely manipulated specific linguistic variables.^12^ Language is an inherent aspect of neurotypical cognitive functioning in humans, and although FTD remains conceptualized as a problem of ‘thought’, it may involve a decline in aspects of cognition inherently linked to language function. There is evidence for a strong association between language impairment and cognitive decline in FTD,^12–17^ and some authors have argued that all SZ is inherently language linked.^18–20^ Enhanced linguistic profiling will support the inroads made into the use of language as a prognostic indicator and marker of disease progression in SZ,^6,8,21–25^ paralleling the critical diagnostic and prognostic role of language in other cognitive disorders, including autism spectrum disorders,^26^ depression,^27^ Huntington’s,^28^ and Alzheimer’s^29^ disease.

## Reference as a linguistic function linked to thought

Referentiality is a key element of all language use, which links it to thought: When talking, we use words to refer to things, events, or people. Consequently, language carries content and informs us about the state of the world. The neurocognitive basis of such reference has been linked to our language capacity,^30,31^ including its non-verbal forms (e.g., pointing) which are closely correlated with language development.^32–34^ Referentiality in language is never solely a lexical property (i.e., a property of words in isolation). Words occurring on their own, e.g., HOUSE, BEAR, WIN, or CUP, do not refer to any particular object: they capture general classes of things and can become referential only when embedded in noun phrases (NPs), which are in turn embedded in utterances.^30^ NPs are grammatical configurations which contain a grammatical function word (*the*, *a*) together with one or more content words providing a description of the referent (*man with a hat*, *red car*), or else consist of pronouns (*she*, *this*) in isolation. Reference to individuals can firstly be ‘generic’, when no particular individual(s) is (are) singled out, as in the sentence *I like* *dogs* (generic NP underlined). Secondly, it can be ‘indefinite-quantificational’, as in *There was* *a dog*, where no specific already known dog is referenced. Thirdly, it can be ‘definite’, as in *I saw* *the dog*, where there is such specificity, or, finally, deictic, as in *I saw* *this dog*. We here group the first two forms of reference as ‘indefinite’ and the last two as ‘definite’. Clinical descriptors of language in FTD such as ‘poverty of content’ or ‘vagueness’ may suggest a problem with definiteness of reference.

There is considerable evidence for referential dysfunction in thought-disordered speech as identified under such labels as discourse cohesion^10,11^ or communication disturbance, in both patients with SZ and their relatives.^35,36^ Misuse of pronouns in FTD has been particularly highlighted in these studies, given their natural impact on discourse cohesion. However, to our knowledge, this dysfunction has neither been profiled from a linguistic point of view, nor across the whole spectrum of NP types. Only such profiling will allow identification of the linguistic or neurocognitive basis of the identified referential dysfunction.

## Syntactic complexity and its significance for FTD

Previous studies of syntactic complexity in SZ^9,15,37^ have tended to explore this issue in SZ generally, and, hence, there is little evidence of a pattern of syntactic complexity specific to FTD. There is evidence that syntactic errors in speech may characterize patients with SZ generally as compared to healthy controls,^3,16^ but other reports indicate that syntactic disturbance may specifically characterize FTD.^4,38,39^ To address this issue, there is a need to decompose the composite variables of structural syntactic complexity used in previous studies,^7,9^ which are often generated from different grammatical construction types (e.g., coordinated, subordinated, and relative clauses along with passives and adjunct clauses). These, however, can play very different linguistic and cognitive roles and carry different kinds of meaning and propositional complexity. For example, embedded (‘complement’) clauses (e.g., *he liked her* in *Mary thought he liked her*) codify mental state contents corresponding another person’s (*Mary’s*) representation of the world. Such constructions thus play an important meta-representational role, as also suggested by correlations between complement clauses and success on theory of mind tasks.^33^ How such syntactic and other linguistic measures relate to non-linguistic cognitive ones is thus an important issue. As of now, some linguistic studies have not employed neuropsychological measures,^9^ while others have not reported any measures of general IQ,^7^ or have only estimated pre-morbid IQ.^15^

## Current study

Here we aimed to profile spontaneous speech in FTD at a fine-grained grammatical level, seeking to differentiate linguistic variables that identify FTD. Three broad linguistic domains were distinguished to capture speech dysfunction: (1) Referential anomalies in the use of NPs, subdivided into three finer distinctions, namely vague and unclear references, third-person anaphor anomalies, and ‘general’ referential anomalies not falling under the two previous types. We also annotated whether referential anomalies occurred in a definite or an indefinite NP, where ‘definite’ comprised all forms of nominal reference requiring specificity, including most uses of *the* + NP, deictic NPs (*this man*), and pronouns (see Supplementary Table 1); (2) syntactic complexity as measured through the number of dependents and embedded clauses per utterance; and (3) syntactic errors (e.g., agreement violations, tense violations, missing/wrong dependents, truncated utterances, and other syntactic violations) (see Supplementary Table 1). We hypothesized that referential anomalies in the use of NPs, along with other linguistic variables measuring the complexity of meaning arising at a syntactic level, could distinguish participants with SZ from those without SZ (first-degree relatives (FDR), and neurotypical controls (NC)), and those with FTD (SZ + FTD) from those without FTD (SZ−FTD). Based on previous literature and theoretical considerations reviewed above, we specifically hypothesized:

H1: SZ + FTD speech would exhibit more referential anomalies than any other group, with FDRs also differing in this regard from neurotypical controls.

H2: SZ + FTD would produce fewer definite NPs.

H3: SZ + FTD would produce the most referential anomalies in definite NPs than any other group.

H4: SZ + FTD and SZ−FTD would produce speech of lesser syntactic complexity as measured by number of grammatical dependents and embedded clauses.

H5: SZ + FTD and SZ−FTD would produce more syntactic errors than FDRs and NCs.

We also explored associations between linguistic measures and general cognition (IQ) and age, and used regression analysis to determine which linguistic variables best predicted group.

## Results

### Referential anomalies

**Fig. 1:**
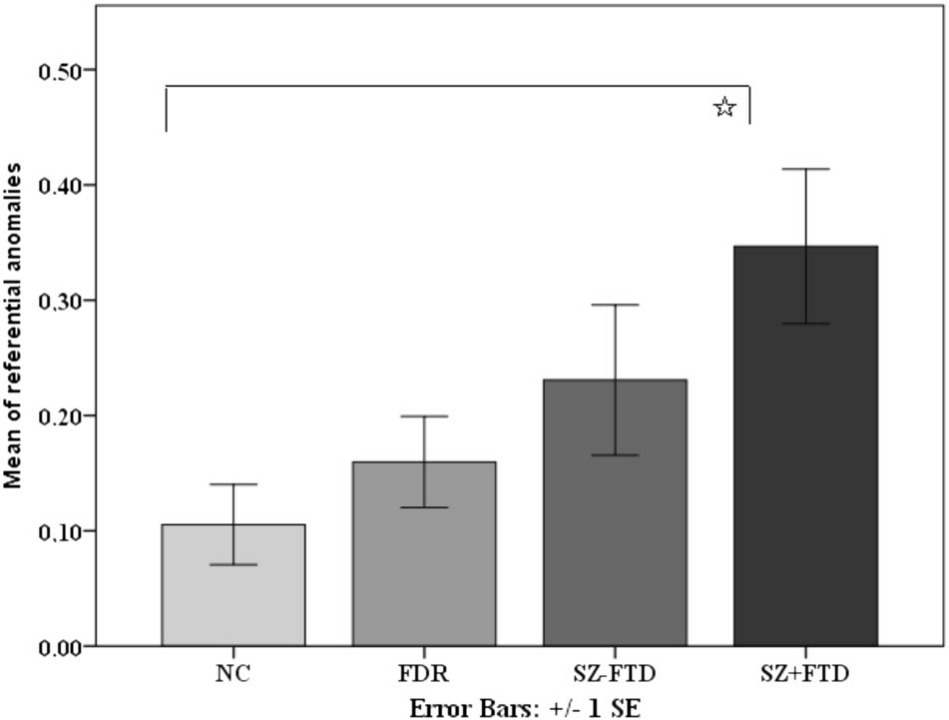
Means of all referential anomalies across neurotypical controls (NC), first-degree relatives (FDR), and participants with (SZ + FTD) and without thought disorder (SZ−FTD). *Pairwise comparisons were significant after Bonferroni correction

There was a group effect on referential anomalies (Supplementary Table 2). Pairwise comparisons reached significance only in the comparison between NC and SZ + FTD, *p* = 0.003 (Fig. 1 and Supplementary Table 3).

### Quantitative production of NP types (disregarding anomalies)

**Fig. 2:**
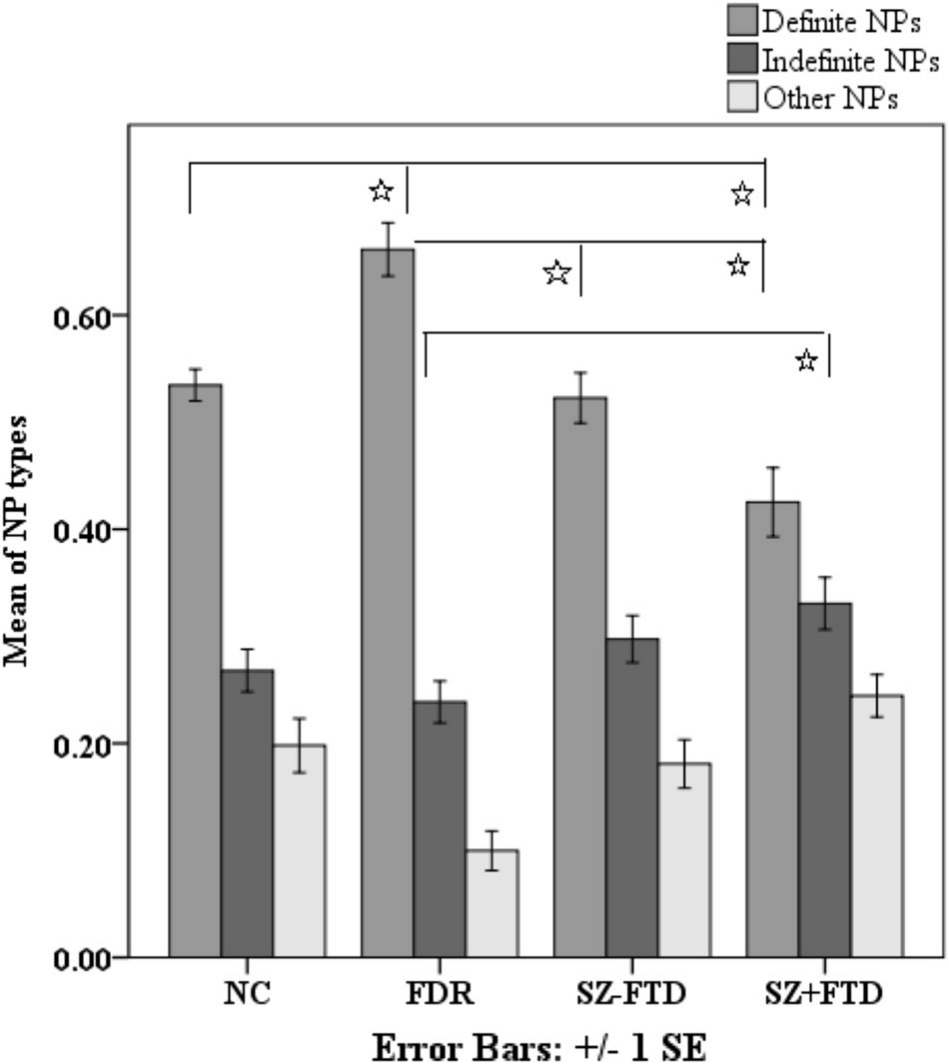
Means of definite, indefinite, and ‘other’ noun phrases [disregarding anomalies] across neurotypical controls (NC), first-degree relatives (FDR), and participants with (SZ + FTD) and without thought disorder (SZ−FTD). *Pairwise comparisons were significant after Bonferroni correction

The SZ + FTD group had the lowest proportion of definite NPs while FDRs had the highest (Fig. 2 and Supplementary Table 4 for definite NPs/total NPs [disregarding anomalies]). Pairwise comparisons were significant between SZ + FTD and both non-clinical groups. However, while SZ−FTD produced more definite NPs than SZ + FTD, the difference did not survive Bonferroni correction. FDRs produced significantly more definite NPs [disregarding anomalies] than all other groups. With regard to indefinite NPs, SZ + FTD showed the highest proportion of use with differences reaching significance in the comparison with FDR (Fig. 2 and Supplementary Table 4 for indefinite NPs/total NPs [disregarding anomalies]).

### Referential anomalies by NP type

**Fig. 3:**
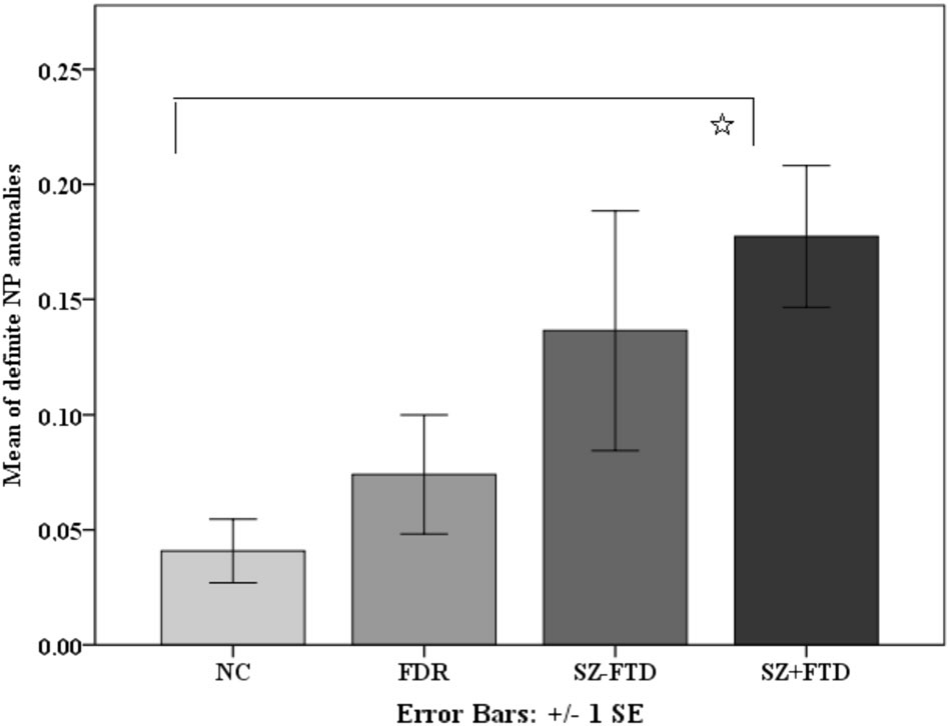
Means of definite NP anomalies in definite NPs across neurotypical controls (NC), first-degree relatives (FDR), and participants with (SZ + FTD) and without thought disorder (SZ−FTD). *Pairwise comparisons were significant after Bonferroni correction

In terms of referential anomalies in NP types, a Kruskal–Wallis test showed a significant group effect for anomalies that occurred in definite NPs only (*p* = 0.018) (Supplementary Table 2). Mann–Whitney *U* test comparisons with Bonferroni corrections showed that differences between NC and SZ + FTD were driven by definite NP anomalies (Fig. 3 and Supplementary Table 4).

### Types of fine-grained referential anomalies

**Fig. 4:**
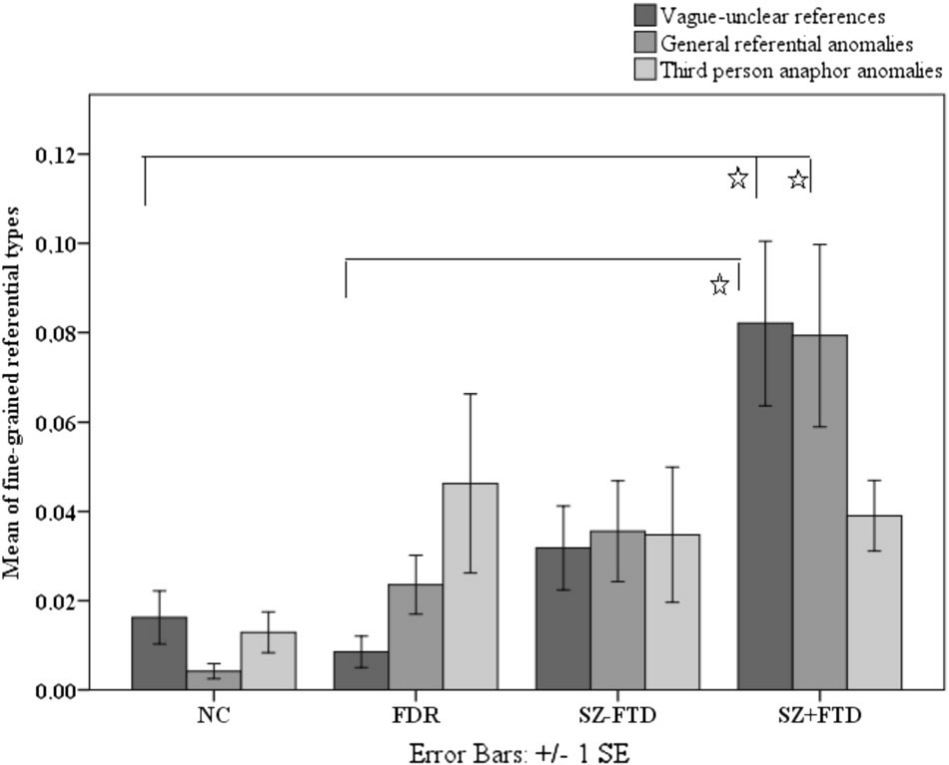
Means of fine-grained referential types across neurotypical controls (NC), first-degree relatives (FDR), and participants with (SZ + FTD) and without thought disorder (SZ−FTD). *Pairwise comparisons were significant after Bonferroni correction

Regarding the more fine-grained sub-classification of referential anomalies, SZ + FTD produced a larger proportion of vague-unclear references than non-clinical groups (*p* = 0.006) (Supplementary Table 5). Kruskal–Wallis did not show a significant group effect for third-person anaphor anomalies (*p* > 0.05) (Supplementary Table 2). Other referential anomalies termed ‘general’ distinguished the groups, but after Bonferroni correction such anomalies were only more frequent for SZ + FTD than NC (*p* = 0.001) (Fig. 4).

### Syntactic complexity and syntactic errors

Groups did not differ in terms of syntactic errors (Supplementary Table 2). Ratios of number of dependents and embedded clauses were significantly smaller in SZ + FTD than in both NC and FDR (Supplementary Tables 6 & 7) (number of dependents: *p* = 0.007; embedded clauses: NC vs. SZ + FTD: *p* = 0.003; FDR vs. SZ + FTD: *p* = 0.022), while the two SZ groups did not significantly differ in this respect (all *p*-values > 0.05). Groups did not differ in their production of syntactic errors (Supplementary Tables 2 & 8).

### Correlations between linguistic variables and Wechsler Abbreviated Scale of Intelligence IQ score and age

After Bonferroni adjustment, a moderate negative association was seen between IQ and total referential anomalies (*r* = −0.424, *p* = 0.001). The association between IQ and total referential anomalies was stronger than with types of NPs with/without anomalies (Referential anomaly occurring in definite NPs *r* = −0.272, *p* = 0.002; Referential anomaly occurring in indefinite NPs: *r* = −0.352, *p* = 0.001; Definite NPs: *r* = 0.304, *p* = 0.001; Indefinite NPs: −0.300, *p* = 0.001) (Supplementary Table 9). There also were positive linear correlations between IQ score and syntactic complexity (IQ with number of dependents: *r* = 0.370, *p* = 0.002; IQ with embedded clauses: *r* = 0.364, *p* = 0.002). The associations between age, on the one hand, and total referential anomalies, syntactic complexity, and IQ, on the other hand, were all non-significant: age with referential anomalies: *r* = 0.127, *p* = 0.166; age with number of dependents: *r* = −0.086, *p* = 0.292; age with embedded clauses: *r* = −0.072, *p* = 0.292; age with IQ: *r* = 0.027, *p* = 0.419.

### Linguistic variables as predictors of group

We explored which linguistic variables—referential anomalies, syntactic complexity, and definite NPs disregarding anomalies—predicted Group (Supplementary Table 10). Referential anomalies (*χ*^2^ = 5.522, *p* = 0.137) did not predict Group and were excluded in the model. Production of definite NPs (disregarding anomalies) and number of dependents, however, had significant main effects on the comparisons of SZ + FTD with NC and FDR (definite NPs: *χ*^2^ = 30.584, *p* = 0.001; dependents: *χ*^2^ = 130.866, *p* = 0.001). The proportions of definite NPs (NC vs. SZ + FTD: Exp (*B*) = 131736; FDR vs. SZ + FTD: Exp (*B*) = 7.377) and the number of dependents (NC vs. SZ + FTD: Exp (*B*) = 25.923; FDR vs. SZ + FTD: Exp (*B*) = 37.501) were smallest in SZ + FTD. Production of definite NPs significantly distinguished SZ−FTD from SZ + FTD, (Exp (*B*) = 110.43, *p* = .038).

## Discussion

H1 and H3 hypothesized that SZ + FTD would produce more referential anomalies than any other group (H1), and the same would be true for referential anomalies in definite NPs specifically (H3). Both hypotheses were partially supported: SZ + FTD produced significantly more referential anomalies than NC, but comparisons with other groups did not reach significance, although group means showed a progression from NC to FDR to SZ−FTD to SZ + FTD (Fig. 1 and Supplementary Table 2). SZ + FTD also produced significantly more anomalies in definite NPs than NC and the comparison to FDR was close to significance (*p* = 0.009) at the corrected significance threshold of *p* = 0.008. (see Fig. 2 and Supplementary Table 4). However, there was no difference between the two SZ groups. This pattern suggests that referential anomalies may represent a marker of FTD, discriminating them from the NC group. Future investigations might increase the size of language samples or expand participant numbers to explore their potential specificity in identifying FTD.

H2 predicted that SZ + FTD would produce fewer definite NPs (disregarding anomalies). This was supported, as SZ + FTD produced the fewest definite NPs (see Fig. 2 and Supplementary Table 4) and significantly differed from both NC and FDR. However, after Bonferroni correction, SZ + FTD did not significantly differ from SZ−FTD. This pattern shows that even when disregarding referential anomalies, definiteness in reference is a linguistic signal that distinguishes patients with FTD but not without FTD from non-clinical controls. Further in line with this conclusion, SZ + FTD produced significantly more vague and unclear references than both NC and FDR (see Fig. 3 and Supplementary Table 5), which signals problems with definiteness and specificity as well. Moreover, when checking for predictability of group membership from linguistic variables, definite NPs were significant predictors, and distinguished SZ−FTD from SZ + FTD. We conclude that definiteness could be an important and more specific linguistic index of FTD, linked to specific grammatical patterns and lying at a junction where thought and language connect.

Interestingly, the FDR group exhibited an idiosyncratic and unpredicted behavior. They did not differ significantly from NCs in terms of either referential anomalies or syntactic complexity, but produced significantly more definite NPs (disregarding anomalies) in relation to all other groups including NC (Supplementary Table 4). In this regard, they exhibited the exactly opposite pattern from SZ + FTD, producing the most definite and fewest indefinite NPs, while SZ + FTD produced the fewest definite and most indefinite NPs. Since of 15 FDRs, only four scored above one in the CD score, with three scoring a two and only one scoring a three, it seems unlikely that the presence of FTD in the FDR group would relate to this result. Consistent with this, the effect went into the opposite direction than in the FTD group, who *under*-produced definites. This pattern is not consistent with previous studies that noted linguistic differences in FDRs captured as ‘referential communication failures’,^35,36^ which would have predicted differences in referential anomalies in our study in the FDR group. This inconsistency informs the question of which linguistic variables can signal vulnerability and likely reflect differences in an annotation process centered on linguistic variables rather than communication failures in a broader sense.

H4 was also partially supported: The SZ + FTD group exhibited significantly lower syntactic complexity than both non-clinical groups, while SZ−FTD again did not differ significantly from these groups, or from SZ + FTD. Interestingly, H5 was not supported: syntactic errors did not differentiate groups. Both findings contrast with earlier findings that have reported more errors and reduced levels of speech complexity in people with SZ in general.^5,7–9,16^ With regards to syntactic complexity, this difference is likely due to the use in the present study of more specific (rather than composite or more generic) linguistic measures. The present results suggest that syntactic complexity effects are again only seen when comparing controls to patients with FTD, but not without FTD. More surprising is that syntactic errors did not distinguish any groups, but H5 needs to be tested further with larger samples and longer narrative tasks. Our results are consistent with previous linguistic studies of reference in FTD^10,11^ that have highlighted problems with cohesion and, within the class of cohesion devices, pronouns, which often fall into the class of definite NPs and function anaphorically (i.e., linking up with previously established referents). However, since participants with FTD produced more anomalies in definite NPs and less definite NPs (disregarding anomalies) compared with both NC and FDR, their difficulty with pronouns may be indicative of a deeper and more general problem with referentiality and definiteness. Moreover, in the finer-grained analysis of referential anomalies, third-person anaphor anomalies did not reach significance, while both vague and unclear references and general referential anomalies did, which suggests the problem is not simply one of anaphoricity or ‘discourse cohesion’. This should be tested in future work and in other languages that differ in their repertoire of function words involving in realizing definiteness (e.g., languages that lack articles such as *the* and *a*).

A referential problem affecting definiteness is part of a semantic impairment, since it concerns meaning. But they are not dimensions relating to lexical-semantic representation or semantic memory directly: they concern meaning in its referential and contextual use, and at a grammatical level of organization. Any conceptual and empirical relationships, if any, between well-noted memory impairments^1,4,16,36,40,41^ and our more direct linguistic measures in the domain of referentiality and complexity are of considerable interest for future work.

Normal referencing is an aspect of normal cognitive functioning and manifest in all normal language use. It is not specific to ‘language’ in any sense that could be separated from ‘thought’, since whenever language is used referentially, thought is necessarily expressed in it. This would make us expect that particularly a referencing deficit could be closely related to measures of cognition at large. In our sample, the two clinical groups did not significantly differ in terms of either their estimated pre-morbid (National Adult Reading Test (NART)) or current (Wechsler Abbreviated Scale of Intelligence (WASI)) IQ (Table 1), but clinical groups were significantly different from non-clinical groups in current IQ, which as noted is consistent with previous studies involving patients with SZ, particularly with FTD.^42–44^ There were positive linear correlations between IQ score and syntactic complexity in the moderate range (number of dependents: *r* = 0.370; embedded clauses: *r* = 0.364), and weak to moderate correlations in referential anomalies (referential anomalies in definite NPs: *r* = −0.272; total referential anomalies: *r* = −0.424,). IQ thus significantly relates to the patterns we found, consistent with the suggestion that language as measured by our variables and neurotypical cognition are linked, but it also leaves much variance unexplained. Regardless of this relation, this study documents a hitherto unknown and differentiated pattern on how linguistic variables do or do not distinguish groups, which illuminates language and SZ alike.

**Table 1:** Demographic and clinical data (mean and standard deviation) for neurotypical controls (NC), first-degree relatives (FDR), and participants with (SZ + FTD) and without (SZ−FTD) formal thought disorder

	NC (*n* = 15)	FDR (*n* = 15)	SZ + FTD (*n* = 15)	SZ−FTD (*n* = 15)	F(df)P^a^ (*n* = 15)
Mean age (years)	45 (13.0)	45 (13.0)	50 (14.6)	38 (7.3)	2.4 (3,56) 0.080
Sex #male	7	8	13	10	
Years of education	16 (3.6)	17 (4.1)	15 (3.6)	13 (4.0)	3.0 (3,56) 0.038
NART	109 (8.7)	101 (12.2)	96 (14.2)	95 (14.0)	4.1(3,55) 0.010
IQ*	107 (8.5)	103 (9.9)	80 (15.7)	87 (19.9)	12.0(3,56) < 0.0005
Illness duration (months)	None	30 (80.4)	232 (149.3)	199 (88.6)	
PANSS item 2	1(0)	1.3(0.6)	5.2(0.9)	1.5(0.6)	
PANSS positive subscale	8.2(0.9)	12 (5.9)	30 (5.0)	21 (4.6)	
PANSS negative subscale	9 (1.4)	12 (6.1)	27 (7.7)	20 (6.8)	
PANSS General Psychopathology Subscale	23 (5.0)	29 (10.2)	39 (16.7)	47 (8.6)	
PANSS total	40 (5.8)	52 (21.1)	74 (33.9)	88 (16.3)	

NART refers to the IQ estimate generated from the National Adult Reading Test.^51^ IQ* refers to the Wechsler Abbreviated Scale of Intelligence (WASI) full scale IQ4^52^

^a^One-way ANOVA

An alternative to the view that reference is inherently language-related is that atypical forms of reference could logically be the expression of cognitive impairment through a language-independent cognitive system. Support for this would be evidence for grounding of referential capacity in such an independent system. However, reference, even in its non-linguistic forms (e.g., declarative index-finger pointing), is widely argued to be developmentally and phylogenetically related to language.^30,32,34^ Moreover, this study shows that it is specifically definiteness in reference that is affected in FTD, and definiteness maps onto specific grammatical configurations rather than others, and is never merely lexical, exhibiting linguistic specificity in this sense. If only for parsimony, the idea that language itself is the cognitive system responsible for reference, with a referential disturbance grounded in language dysfunction, should therefore be explored.

A number of factors could have influenced the present results, which were only partially controlled for. In particular, FTD is not static and can change over time depending on a number of conditions, including context, emotional states, or the content of speech. While steps were taken to minimize these effects (e.g., we chose a non-personal speech elicitation task, and the participants spent a lot of time with the research team and in the testing environment before recordings were made, and as such were habituated to the test procedure), such effects cannot be ruled out. Other factors that could have been considered are the patients’ living arrangements, which could influence daily sociability and communication and possibly language, though we know of no independent evidence that a relation between these factors and the specific linguistic variables tested in this study would be expected. Moreover, living arrangements were highly variable in both patient groups.

In summary, this study has identified referentiality as linked to grammar and particularly definiteness, along with specific measures of grammatical complexity, as linguistic markers that distinguish FTD (but not SZ at large) from non-clinical controls. Future work should further investigate specificity of our measures to patients with FTD as opposed to patients without, which would have important implications for understanding and measuring FTD and mapping it onto cognitive processes and brain pathways. Using standardized aphasia-based measures, both SZ and FTD have long been linked to language impairment as seen in aphasia.^14,16,17,45^ However, direct comparisons of referential impairment in SZ and aphasia, using fine-grained grammatical measures, have not been carried out, to our knowledge. Future work should deepen our insights into this dysfunction in the speech of patients with SZ as compared to that of patients with aphasia and at the neural level.^45,46^ Such work will provide important targets for future clinical, natural history, and neurobiological studies.

## Methods

### Participants

NRES Committee North East - Newcastle & North Tyneside 2 approved our human study protocol. All participants provided written, informed consent. Fifteen participants with FTD (SZ + FTD) and 15 without (SZ−FTD) were recruited from a UK secondary care mental healthcare trust (Northumberland, Tyne and Wear (NTW) NHS Foundation Trust). All participants with SZ met DSM-IV diagnostic criteria and scored at least 60 on the Positive and Negative Symptom Scale for Schizophrenia (PANSS).^47^ This cut-off point was selected to generate a sample who were symptomatic at least at the level found in stable outpatients.^48^ The PANSS was completed by trained and experienced raters (M.D. and H.S.) and inter-rater reliability checks were undertaken to ensure internal consistency of the ratings, with the help of senior academic psychiatrists (D.T. and S.W.).

Fifteen NC participants were recruited via an advert placed in hospital and university buildings. Fifteen FDRs of people with SZ were also recruited via carers groups and via patients in NTW trust and study participants. Exclusion criteria were substance dependence or abuse, pervasive developmental disorder interfering with language skills, severe epilepsy, significant head injury, stroke, and brain tumor in all participants, and past or current psychotic disorder in NC participants and FDRs. Participants with SZ were dichotomized on the basis of their score on question 2 (‘Conceptual Disorganization’, CD) of the PANSS. Those who scored at least four (which equates to an anchor of at least ‘moderate’ on this item) were categorized as SZ + FTD, those who scored three (‘minimal’) or less were categorized as SZ−FTD. FDRs also completed the PANSS. Of 15 FDRs, 11 scored one on the CD scale (=no conceptual disorganization), three scored two (=questionable pathology), one scored three (=circumstantial, tangential or paralogical thinking, difficulty in directing thoughts towards a goal, some loosening of associations evidenced under pressure).

All subjects were native speakers of English and willing to have their interviews audio recorded for the purposes of linguistic analysis. Table 1 summarizes the demographic and clinical data.

Four of the SZ + FTD participants were taking clozapine, six olanzapine, one amisulpride, two haloperidol, one aripirazole, one risperidone, and two were prescribed a first-generation depot. In addition, two were taking Selective serotonin reuptake inhibitors (SSRIs), one trazodone, two diazepam, one zopiclone, one codeine, and three procyclidine. Of the SZ−FTD group, eight were prescribed clozapine, two olanzapine, three amisulpride, one sulpiride, three aripiprazole, one quetiapine, one a first-generation depot, one an SSRI, one venlafaxine, one mirtazepine, one temazepam, one zopiclone, one pregabalin, and two sodium valproate. Of the FDRs, one was taking an SSRI, one venlafaxine, one thyroxine, one propranolol, and one codeine. Of the neurotypical subjects, none were taking psychoactive drugs.

SZ−FTD subjects were significantly younger than SZ + FTD (*p* = 0.009) (Supplementary Table 11). They were significantly less educated than NC and FDR (*p* = 0.024; *p* = 0.016). Total length of illness did not correlate with our measure of FTD (question 2 on the PANSS) (*ρ* = 0.056,), nor did it correlate with the relevant PANSS sub-scale (positive symptoms (*ρ* = −0.046) nor with PANSS total score (*ρ* = −0.097); in either the FTD sub-group, or the combined group of patients with SZ; using Spearman’s (*ρ* < 2.8, *p* > 0.15).

The two clinical groups were not significantly different in years of education (*p* > 0.05). SZ−FTD and SZ + FTD were matched on WASI IQ score (*p* > 0.05) (Supplementary Table 11), but both clinical groups had significantly lower IQ scores than NC and FDR. This is consistent with previous studies involving patients with SZ, particularly with FTD.^42,43^

### Linguistic testing

Samples of connected speech were collected while participants told the story depicted by an eight-picture comic strip in which a cat steals a fish intended for dinner guests.^49^ The strip contains only a few words (specifically, the story title: ‘The dinner party’; a guest note: ‘8 PM Friday dinner with Smiths,’ and a shop sign: ‘Fish and Chips’). All participants were given the same instruction: ‘Together, these pictures create a story and please tell me the story and everything you see going on in your own words.’ All pictures were in view while participants discussed them. Audio files were anonymized and coded so that raters were blind to diagnosis/group membership. All audio files had good sound quality and were transcribed by three native speakers of English, who were blind to diagnosis. Any word or utterance disagreements were discussed among transcribers until resolved. In addition, the study’s first and second authors, who were never in contact with participants and blind to diagnoses, annotated all participants’ narrations based on the annotation scheme described below. During the annotation process, first, second, and last authors discussed and reached agreement on all questionable or disagreed-upon cases.

### Annotation and linguistic variables

In this study, an utterance was defined as a (self-standing) grammatically independent unit of discourse providing new information. A clause was defined as a configuration with a subject and predicate (usually a verb). Variables annotated in this study are exemplified below:

#### *Referential anomalies*

Vague-unclear references: ‘There is a man phoning a man and *he* is making an appointment to come and have dinner with *him’*—difficult to disambiguate *he*/*him*.

Third-person anaphor anomaly: ‘Resk saves the day. *They* come home’—the anaphoric pronoun *they* is used, but only one character ‘comes home’ (the other three never left).

General-referential anomaly: ‘He is cracking someone’s head on the dance floor’—objects/events are referenced that do not exist in the cartoon.

Referential anomaly occurring in definite NP: ‘It is *the two people* from picture number four’—a more specific NP (e.g., ‘the couple’) is expected as these main story characters have been present throughout the story.

Referential anomaly occurring in indefinite NP: ‘*Someone* is crying’—the indefinite NP ‘someone’ is used where a definite NP (e.g., *she*) would be expected, since the referred character has been already introduced.

#### *Noun phrase (NP) types (disregarding anomalies)*

Definite NP: ‘*The other guy* is doing the dishes’—the NP refers to a specific, previously mentioned individual.

Indefinite NP: **‘***A funny looking cat* is sitting under the table’—NP introduces a new referent into the narrative.

#### *Syntactic complexity*

Number of dependents: ‘They *have realized* that the cat *has eaten* the fish’—there are four dependents: Two depend on the head *have realized* (‘they’, ‘that the cat has eaten the fish’), and the other two depend on the head *has eaten* (‘the cat’, ‘the fish’).

Number of embedded clauses: ‘And I would guess the gentleman is inviting his boss around for dinner to impress him.’ There are two embedded clauses: a complement clause (‘the gentleman is inviting …’), and an adjunct clause (‘to impress him’).

#### *Syntactic errors (i.e., agreement violations, tense violations, missing/wrong dependents, truncated utterances, and other syntactic errors)*

Agreement violation: “Err obviously the guy: the younger one and his wife is slaving away in the kitchen.” Singular verb ‘is’ is used for plural subject.

Truncated utterance: “Erm. They send.” The utterance is truncated after ‘send’.

### Data analysis

Statistical analysis was divided into three stages. First, referential anomalies, the production of definite/indefinite NPs, syntactic complexity, and syntactic errors were compared across groups (Supplementary Table 2). To control for the overall quantities of speech produced by each participant, referential anomalies were converted into ratios by dividing the total number of anomalies by the total number of produced utterances. Next, we determined whether groups differed in terms of quantitative production of different types of NPs disregarding anomalies (that is, NPs were counted regardless of whether they were anomalous), namely: (1) Definite NPs; (2) Indefinite NPs; and (3) ‘Other’ NPs not subsumable under the previous two categories. “Other” NPs include: expletives (e.g., *it*, *there*, etc.), which do not convey normal referential meaning, 1st and 2nd Person pronouns, which carry personal meaning not relevant to the cartoon task, and proper names, which have a special status and do not involve determiners (Supplementary Table 1; Tables 12 and 13 for excluded NPs). Definite, indefinite NPs, and ‘other’ NPs were counted and divided by the total number of NPs produced. We then tested whether the proportion of referential anomalies occurring in definite NPs was different across groups, and analogously for referential anomalies in indefinite NPs. To this end, anomalies in definite and indefinite NP were divided by the number of definite and indefinite NPs, respectively, to obtain definite and indefinite error ratios. Finally, a more fine-grained subdivision of referential anomalies was tested for differences across groups: vague unclear references, third-person anaphor anomalies, and ‘general’ referential anomalies; all of these were divided by the total number of NPs produced. Given that our predictions were in one direction (e.g., more anomalies in definite NP for clinical groups than NC and FDR), we report 1-tailed significance values.

For syntactic complexity, ratios were as follows: (a) total number of dependents to total number of utterances, and (b) embedded clauses to total number of utterances. In addition, the total number of syntactic errors (e.g., tense agreement, truncated utterances, and missing/or wrong dependents) was divided by the total number of utterances.

Where variables were normally distributed, a univariate analysis of normal general linear model (GLM) with Bonferroni post hoc test was applied. For the remaining variables, we ran non-parametric tests. Mann–Whitney *U* pairwise comparisons were carried out when Kruskal–Wallis showed a significant effect of group, and Bonferroni correction was applied.

In stage two, correlations were computed between WASI IQ score, age, and ratios of each type of linguistic variable with/without anomalies. Pearson correlation was conducted for normally distributed variables and Kendall rank correlation was computed for the variables not normally distributed.

In the third stage, we ran multinominal regression to explore whether our main linguistic variables (i.e., referential anomalies, syntactic complexity, and definite NPs [disregarding anomalies]) would be the best predictors of Group. In order to avoid multicollinearity, fine-grained referential anomalies and errors in definite NPs were not included in the model. Variables for which Kruskal–Wallis did not show a significant group effect were excluded from the model. Group was a dependent variable, and referential anomalies, definite NPs disregarding anomalies, and dependents were added to the model as independent variables. A stepwise regression, with backward method, was run in SPSS.

## Electronic supplementary material

Supplementary materials

## Data Availability

The datasets generated during and/or analyzed during the current study are available in OFSHOME repository, [PaLS: https://osf.io/3fksh/] (ref. ^50^).

## Electronic supplementary material

**Supplementary information** accompanies the paper on the *npj Schizophrenia* website (10.1038/s41537-018-0061-9).

## References

[1] Barrera A, McKenna P, Berrios GE (2005). Formal thought disorder in schizophrenia: an executive or a semantic deficit?. Psychol. Med..

[2] McKenna, P. J. & Oh, T. M. *Schizophrenic Speech: Making Sense of Bathroots and Ponds that Fall in Doorways*. (New York, NY, US: Cambridge University Press, 2005).

[3] Stirling J, Hellewell J, Blakey A, Deakin W (2006). Thought disorder in schizophrenia is associated with both executive dysfunction and circumscribed impairments in semantic function. Psychol. Med..

[4] Tan EJ, Neil E, Rossell SL (2015). Assessing the relationship between semantic processing and thought disorder symptoms in schizophrenia. J. Int. Neuropsychol. Soc..

[5] DeLisi LE (2001). Speech disorder in schizophrenia: review of the literature and exploration of its relation to the uniquely human capacity for language. Schizophr. Bull..

[6] Fraser WI, King KM, Thomas P, Kendell RE (1986). The diagnosis of schizophrenia by language analysis. Br. J. Psychiatry.

[7] Marini A (2008). The language of schizophrenia: an analysis of micro and macrolinguistic abilities and their neuropsychological correlates. Schizophr. Res..

[8] Morice R, McNicol D (1986). Language changes in schizophrenia: a limited replication. Schizophr. Bull..

[9] Tavano A (2008). Specific linguistic and pragmatic deficits in Italian patients with schizophrenia. Schizophr. Res..

[10] Rochester, S. *Crazy Talk: A Study of the Discourse of Schizophrenic Speakers*. (New York, NY, US: Springer Science & Business Media, 2013).

[11] Harvey PD (1983). Speech competence in manic and schizophrenic psychoses: the association between clinically rated thought disorder and cohesion and reference performance. J. Abnorm. Psychol..

[12] Wensing T (2017). Neural correlates of formal thought disorder: an activation likelihood estimation meta-analysis. Hum. Brain Mapp..

[13] Kim SJ (2015). The relationship between language ability and cognitive function in patients with schizophrenia. Clin. Psychopharmacol. Neurosci..

[14] Landre NA, Taylor MA, Kearns KP (1992). Language functioning in schizophrenic and aphasic patients: cognitive and behavioral neurology. Neuropsychiatry, Neuropsychol. Behav. Neurol..

[15] Moro A (2015). Detecting syntactic and semantic anomalies in schizophrenia. Neuropsychologia.

[16] Oh TM, McCarthy RA, McKenna PJ (2002). Is there a schizophasia? A study applying the single case approach to formal thought disorder in schizophrenia. Neurocase.

[17] Rodriguez-Ferrera S, McCarthy RA, McKenna PJ (2001). Language in schizophrenia and its relationship to formal thought disorder. Psychol. Med..

[18] Crow TJ (2008). The ‘big bang’ theory of the origin of psychosis and the faculty of language. Schizophr. Res..

[19] Hinzen W, Rosselló J (2015). The linguistics of schizophrenia: thought disturbance as language pathology across positive symptoms. Front. Psychol..

[20] Hinzen W, Rosselló J, McKenna P (2016). Can delusions be understood linguistically?. Cogn. NeuroPsychiatry.

[21] Bedi G (2015). Automated analysis of free speech predicts psychosis onset in high-risk youths. npj Schizophrenia.

[22] Bearden C, Wu K, Caplan R, Cannon T (2011). Thought disorder and communication deviance as predictors of outcome in youth at clinical high risk for psychosis. Am. J. Psychiatry.

[23] Brown M, Kuperberg GR (2015). A hierarchical generative framework of language processing: linking language perception, interpretation, and production abnormalities in schizophrenia. Front. Hum. Neurosci..

[24] Reichenberg A (2002). A population-based cohort study of premorbid intellectual, language, and behavioral functioning in patients with schizophrenia, schizoaffective disorder, and nonpsychotic bipolar disorder. Am. J. Psychiatry.

[25] Rosenstein M, Foltz PW, DeLisi LE, Elvevåg B (2015). Language as a biomarker in those at high-risk for psychosis. Schizophr. Res..

[26] Kuhl PK (2013). Brain responses to words in 2-year-olds with Autism predict developmental outcomes at age 6. PLoS ONE.

[27] Fineberg SK (2015). Word use in first-person accounts of schizophrenia. Br. J. Psychiatry.

[28] Hinzen, W. et al. A systemic linguistic profile of spontaneous narrative speech in pre-symptomatic and early stage Huntington's disease. *Cortex*. 10.1016/j.cortex.2017.07.022 (2017). 10.1016/j.cortex.2017.07.022 PMC5845634 28859906

[29] Ahmed S, High AMF, de Jager CA, Garrard P (2013). Connected speech as a marker of disease progression in autopsy-proven Alzheimer’s disease. Brain.

[30] Hinzen W (2017). Reference across pathologies: a new linguistic lens on disorders of thought. Theor. Linguist..

[31] Hinzen, W. & Sheehan, M. *The Philosophy of Universal Grammar*. (Oxford: Oxford University Press, 2015).

[32] Butterworth, G. in *Pointing: Where Language, Culture, And Cognition Meet* (ed Kita, S.) 9–33 (Lawrence Erlbaum Associates Publishers, Mahwah, NJ, US, 2003).

[33] de Villiers J (2007). The interface of language and theory of mind. Lingua.

[34] Tomasello, M. in *Roots of Human Sociality: Culture, Cognition And Interaction*. (Eds Enfield, N. J. & Levinson, S. C.) 506–524 (Oxford, Berg, New York, 2006).

[35] Docherty NM, Gordinier SW (1999). Immediate memory, attention and communication disturbances in schizophrenia patients and their relatives. Psychol. Med..

[36] Docherty NM (2012). On Identifying the processes underlying schizophrenic speech disorder. Schizophr. Bull..

[37] Condray R, Steinhauer SR, van Kammen DP, Kasparek A (2002). The language system in schizophrenia: effects of capacity and linguistic structure. Schizophr. Bull..

[38] Faber R, Reichstein MB (1981). Language dysfunction in schizophrenia. Br. J. Psychiatry.

[39] Walenski M, Weickert TW, Maloof CJ, Ullman MT (2010). Grammatical processing in schizophrenia: evidence from morphology. Neuropsychologia.

[40] Goldberg TE, Dodge M, Aloia M, Egan MF, Weinberger DR (2000). Effects of neuroleptic medications on speech disorganization in schizophrenia: biasing associative networks towards meaning. Psychol. Med..

[41] Pomarol-Clotet E, Oh TMSS, Laws KR, McKenna PJ (2008). Semantic priming in schizophrenia: systematic review and meta-analysis. Br. J. Psychiatry.

[42] Cuesta MJ, Peralta V (1995). Cognitive disorders in the positive, negative, and disorganization syndromes of schizophrenia. Psychiatry Res..

[43] Dibben CRM, Rice C, Laws K, McKenna PJ (2009). Is executive impairment associated with schizophrenic syndromes? A meta-analysis. Psychol. Med..

[44] O’Leary DS (2000). Cognitive correlates of the negative, disorganized, and psychotic symptom dimensions of schizophrenia. J. Neuropsychiatry Clin. Neurosci..

[45] Halpern H, McCartin-Clark M (1984). Differential language characteristics in adult aphasic and schizophrenic subjects. J. Commun. Disord..

[46] Kuperberg GR, Ditman T, Choi Perrachione A (2018). When proactivity fails: an electrophysiological study of establishing reference in schizophrenia. Biol. Psychiatry.

[47] Kay SR, Fiszbein A, Opler LA (1987). The positive and negative syndrome scale (PANSS) for schizophrenia. Schizophr. Bull..

[48] Opler LA, Malaspina D, Opler MG (2006). Reducing guesswork in schizophrenia treatment. Curr. Psychiatry.

[49] Fletcher M, Birt D (1983). Storylines: Picture Sequences For Language Practice.

[50] Cokal, D. et al. PaLS. Open Science Framework. http://osf.io/3fksh (2018).

[51] Nelson, H. E., & Willison, J. (1991). The national adult reading test (NART): test manual. Windsor, UK:NFER-Nelson.

[52] Wechsler, D. (2011). Wechsler abbreviated scale of intelligence - second edition (WASI-II). San Antonio,TX: NCS Pearson.

