# Supplementary material for: The language profile of formal thought disorder
Source: NPJ Schizophr. 2018 Sep 19;4:18. doi: 10.1038/s41537-018-0061-9 (PMC6145886; doi:10.1038/s41537-018-0061-9)
Supplement: Supplementary file 1 — Supplementary materials [file 41537_2018_61_MOESM1_ESM.pdf]

NP categories	Types	Example
	3rd person definite	*The man* with the hat is my boyfriend.
Definite NPs	3rd person pronominal anaphors	John ran to the fish and chip shop. *He* was not in a very good mood.
	3rd personal pronominal cataphora	*He* is John.
	3rd person deictic	*This lady* is cooking.
Indefinite NPs	2nd person generic	*You* don't urinate in the streets.
	3rd person generic/abstract	*Cats* are my favourite animals.
	3rd person indefinites	*A man* is calling *someone*.
Other NP types	1st person singular/plural	*I* don't like cats.
	2nd person deictic	Why do *you* ask me this question?
	3rd person rigid	*London* is my favourite capital city.
	Expletives	*It* is raining today.

Supplementary Table 1 Categories of noun phrases (NP)

Linguistic variables (ratios)	Model Type	Statistics	p	η^2
Referential anomalies / total utterances	Univariate-GLM	F (3,59) = 4.985	.004*	.21
Definite NPs / total NPs	Kruskal-Wallis	H (3) = 24.678	.001*	.33
Indefinite NPs / total NPs	Kruskal-Wallis	H (3) = 9.226	.026*	.097
Other NPs/total NPs	Kruskal-Wallis	H (3) = 17.150	.001	.022
Anomalies in definite NPs / total definite NPs	Kruskal-Wallis	H (3) = 10.113	.018*	.11
Anomalies s in indefinite NPs / total indefinite NPs	Kruskal-Wallis	H (3) = 6.563	.087	.06
Vague and unclear references / total NPs	Kruskal-Wallis	H (2) = 12.501	.006*	.15
Third-person anaphor anomalies / total NP	Kruskal-Wallis	H (2) = 5.554	> .05	None
General referential anomalies / total NPs	Kruskal-Wallis	H (2) = 12.895	.005*	.16
Dependents / total utterances	Univariate-GLM	F (3,59) = 6.744	.001*	.06
Embedded clauses / total utterances	Univariate-GLM	F (3,59) = 5.659	.002*	.04
Syntactic errors / total utterances	Univariate-GLM	F (3,59) = 1.944	> .05	None

*Mean difference significant at .05 level.

Supplementary Table 2 General statistical significance for possible between-group differences in linguistic variables

	Group	Mean (SD)	Comparisons	Mean difference	SE	p	95% CI Lower Bound	Upper Bound
Referential anomalies/ total number of utterances	NC	0.11 (.13)	NC vs. SZ+ FTD	-0.243	.065	.003*	-0.422	-0.064
	FDR	0.16 (.15)	NC vs. SZ-FTD	-0.157	.065	.116	-0.337	0.215
	SZ-FTD	0.23 (.25)	NC vs. FDR	-0.086	.065	1.00	-0.266	0.092
	SZ+ FTD	0.35 (.26)	FDR vs. SZ+ FTD	-0.156	.065	.122	-0.335	0.227
	-------	-------	FDR vs. SZ-FTD	0.071	.065	1.00	-0.250	0.108
	--------	-------	SZ+ FTD vs. SZ-FTD	0.085	.065	1.00	-0.093	0.264

* The mean difference is significant at the .05 level.

Supplementary Table 3 Comparisons of referential anomalies by neurotypical controls (NC), first degree relatives (FDR), participants with FTD (SZ+FTD), and without FTD (SZ-FTD) in univariate analysis of GLM

Linguistic Variables	Group	Mean (s.d.)	Comparisons	U	Z	Sig. (1-tailed)	Effect size (η^2)	Bonferroni correction
Definite NPs /total NPs (disregarding anomalies)	NC	0.53 (.058)	NC vs. SZ+ FTD	45.00	-2.805	.003*	.26	<.008*
	FDR	0.66 (.098)	NC vs. SZ-FTD	98.00	-.602	.27	None	>.008
	SZ-FTD	0.52 (.090)	NC vs. FDR	36.50	-3.155	.001*	.33	<.008*
	SZ+FTD	0.43 (.124)	FDR vs. SZ+ FTD	20.50	-3.820	.001*	.49	<.008*
			FDR vs. SZ-FTD	29.00	-3.468	.001*	.04	<.008*
			SZ+ FTD vs. SZ-FTD	56.00	-2.351	.010*	.18	>.008
Indefinite NPs / total NPs (disregarding anomalies)	NC	0.27 (.078)	NC vs. SZ+ FTD	65.00	-1.974	.24	.13	>.008
	FDR	0.24 (.076)	NC vs. SZ-FTD	81.50	-.1.288	.089	None	>.008
	SZ-FTD	0.30 (.086)	NC vs. FDR	96.00	-.684	.25	None	>.008
	SZ+FTD	0.33 (.092)	FDR vs. SZ+FTD	47.50	-2.700	.004*	.24	<.008*
			FDR vs. SZ-FTD	63.50	-2.058	.025*	.14	>.008
			SZ+ FTD vs. SZ-FTD	95.50	-.707	.240	None	>.008
Referential anomalies in definite NPs/ total definite NPs	NC	0.04 (.054)	NC vs. SZ+ FTD	33.00	-3.329	.005*	.036	<.008*
	FDR	0.07 (.100)	NC vs. SZ-FTD	92.00	-.879	.190	None	>.008
	SZ-FTD	0.14 (.201)	NC vs. FDR	100.50	-.514	.303	None	>.008
	SZ+FTD	0.18 (.119)	FDR vs. SZ+ FTD	55.50	-2.387	.009*	.019	>.008
			FDR vs. SZ-FTD	103.50	-.386	.300	None	>.008
			SZ+ FTD vs. SZ-FTD	78.50	-1.424	.077	None	>.008

Supplementary Table 4 Pairwise comparisons of neurotypical controls (NC), first degree relatives (FDR), participants with (SZ+FTD), and without Formal Thought Disorder (SZ-FTD) on the use of definite and indefinite NPs without/with anomalies in Mann Whitney U Test

Linguistic Variables	Group	Mean (s.d.)	Comparisons	U	Z	Sig. (1-tailed)	Effect size (η^2)	Bonferroni correction
Vague and unclear references /total NPs	NC	0.02 (.023)	NC vs. SZ+FTD	52.000	2.594	0.006*	.21	<.008*
	FDR	0.01 (.013)	NC vs. SZ-FTD	89.000	1.042	0.149	None	>.008
	SZ-FTD	0.03 (.036)	NC vs. FDR	95.000	-.820	0.206	None	>.008
	SZ+FTD	0.08 (.071)	FDR vs. SZ+ FTD	45.000	2.954	0.002	.26	<.008*
			FDR vs. SZ-FTD	75.000	-1.719	0.04*	.08	>.008
			SZ+ FTD vs. SZ-FTD	64.000	2.063	0.023*	.14	>.008
General referential anomalies /total NPs	NC	0.004 (.006)	NC vs. SZ+FTD	40.000	-3.174	0.001*	.40	<.008*
	FDR	0.02 (.025)	NC vs. SZ-FTD	64.000	-2.187	0.015*	.14	>.008
	SZ-FTD	0.04 (.043)	NC vs. FDR	63.000	-2.230	0.013*	.14	>.008
	SZ+FTD	0.08 (.078)	FDR vs. SZ+ FTD	63.500	-2.071	0.019*	.14	>.008
			FDR vs. SZ-FTD	101.00	-.493	0.207	None	>.008
			SZ+ FTD vs. SZ-FTD	78.000	1.458	.0725	None	>.008
Third-person[1] anaphor anomalies / total NP	NC	0.01 (.017)	NC vs. SZ+ FTD	-------	-------	-------	-------	-------
	FDR	0.05 (.077)	NC vs. SZ-FTD	-------	-------	-------	-------	-------
	SZ-FTD	0.03 (.058)	NC vs. FDR	-------	-------	-------	-------	-------
	SZ+FTD	0.04 (.030)	FDR vs. SZ+ FTD	-------	-------	-------	-------	-------
			FDR vs. SZ-FTD	-------	-------	-------	-------	-------
			SZ+ FTD vs. SZ-FTD	-------	-------	-------	-------	-------

Supplementary Table 5 Pairwise Comparisons of Neurotypical Controls (NC), First Degree Relatives (FDR), Participants with (SZ+FTD), and without Formal Thought Disorder (SZ-FTD) on the Use of Fine-grained Referential Types in Mann Whitney U Test

	Pairwise comparisons	Mean difference	Std. Error	p	95% CI Lower Bound	95% CI Upper Bound
Dependents / total utterances	NC vs. SZ+ FTD	0.793	.231	.007*	0.160	1.425
	NC vs. SZ-FTD	0.511	.231	>.05	-0.121	1.143
	NC vs. FDR	0.000	.231	>.05	-0.631	0.633
	FDR vs. SZ+ FTD	0.792	.231	.007*	0.160	1.425
	FDR vs. SZ-FTD	0.510	.231	>.05	-0.121	1.143
	SZ+ FTD vs. SZ-FTD	-0.282	.231	>.05	-0.914	0.350
Embedded clauses/ total utterances	NC vs. SZ+ FTD	0.255	.068	.003*	0.068	0.443
	NC vs. SZ-FTD	0.137	.068	>.05	-0.049	0.325
	NC vs. FDR	0.047	.068	>.05	-0.139	0.235
	FDR vs. SZ+ FTD	0.207	.068	.022*	0.020	0.395
	FDR vs. SZ-FTD	0.089	.068	>.05	-0.097	0.277
	SZ+ FTD vs. SZ-FTD	0.117	.068	>.05	-0.069	0.305

* The mean difference is significant at the .05 level.

Supplementary Table 6 Comparisons of Syntactic Complexity by Neurotypical Controls (NC), First Degree Relatives (FDR), Participants with FTD (SZ+FTD), and without FTD (SZ-FTD) in Univariate Analysis of GLM

	NC	FDR	SZ-FTD	SZ+FTD
Dependents	2.78 (.447)	2.86 (.881)	2.33 (.533)	2.03 (.268)
Embedded clauses	0.47 (.138)	0.46 (.264)	0.37 (.154)	0.23 (.138)

Supplementary Table 7 Mean of ratio (M) and Standard Deviation (s.d.) for number of dependents and embedded clause

Linguistic Variable	Group	M (s.d.)
Syntactic errors /total utterances	NC	.070 (.068)
	FDR	.071(.064)
	SZ-FTD	.110 (.102)
	SZ+FTD	.114 (.105)

Supplementary Table 8 Mean (M) and Standard Deviation (s.d.) for Syntactic Errors

Linguistic variable	Subcategories	1	2	3	4	5	6	7
Fine-grained referential types	Vague-unclear references	1.00						
	Third-person anaphor anomaly	.285*<br>p = .002	1.00					
	General-referential anomaly	.234<br>p = .010	.265*<br>p = .005	1.00				
	Referential anomaly occurring in definite NP	.465*<br>p = .001	.667*<br>p = .001	.400*<br>p =.001	1.00			
	Referential anomaly occurring in indefinite NP	.435*<br>p = .001	.297*<br>p = .002	.476*<br>p = .001	.435<br>p = .001	1.00		
Noun phrase type	Definite noun phrase	-.218<br>p = .011	-.022<br>p = .408	-.286*<br>p = .001	-.133<br>p = .076	-.217<br>p =.013	1.00	
	Indefinite noun phrase	.243*<br>p = .006	.094<br>p = .162	.328*<br>p = .001	.227<br>p = .008	.346*<br>p = .001	-.421<br>p = .001	1.00
	WASI	-.161<br>p = .046	-.172<br>p = .035	-.418*<br>p = .001	-.272*<br>p = .002	-.352*<br>p = .001	.304*<br>p = .001	-.300*<br>p = .001
	Age	.087<br>p = .183	-.039<br>p = .340	.060<br>p = .263	-.053<br>p = .283	.014<br>p = .442	.036<br>p = .346	.036<br>p = .346

*A Bonferroni correction was applied by dividing the alpha value by the number of comparisons. The significance threshold was set at 0.007.

Supplementary Table 9 Correlations of linguistic variables with WASI IQ and age of neurotypical controls (NC), first degree relatives (FDR), and participants with (SZ+FTD) and without (SZFTD) formal thought disorder.

				95% CI for Odds Ratio		
Groups	*B* (SE)	Wald	*p*	Lower	Odds Ratio	Upper
NC vs. SZ+FTD						
Intercept	-13.394	11.196	.001*	------	------	------
Definite NPs	11.789	4.917	.027*	3.928	131736	4417618
Dependents	3.255	7.708	.005*	2.604	25.923	258.041
FDR vs. SZ+FTD						
Intercept	-23.748	20.332	.001*	------	------	------
Definite NPs	27.327	16.840	.001*	1583371	7.377	3.437
Dependents	3.624	8.200	.004*	3.138	37.501	448.101
SZ-FTD vs. SZ+ FTD						
Intercept	-8.099	3.197	.011*	------	------	------
Definite NPs	9.310	4.316	.038*	1.623	110.43	72033960
Dependents	1.683	2.389	.122	.637	5.383	45.505

Note: R^2 = .574 (Cox & Snell), .61 (Nagelkerke). Model χ^2 = 51.191, p = .001

Supplementary Table 10 Linguistic variables as predictors of group

	Comparisons	t	df	Sig. (2-tailed)
Age	NC vs. SZ+ FTD	.952	28	> .05
	NC vs. SZ-FTD	1.836	28	> .05
	NC vs. FDR	.042	28	> .05
	FDR vs. SZ+ FTD	.952	28	> .05
	FDR vs. SZ-FTD	1.787	28	.085
	SZ+ FTD vs. SZ-FTD	-2.825*	28	.009
Education	NC vs. SZ+ FTD	1.142	28	> .05
	NC vs. SZ-FTD	-2.386*	28	.024
	NC vs. FDR	-.330	28	> .05
	FDR vs. SZ+ FTD	1.403	28	> .05
	FDR vs. SZ-FTD	-2.568*	28	.016
	SZ+ FTD vs. SZ-FTD	-1.324	28	> .05
IQ/WASI	NC vs. SZ+ FTD	-5.763*	28	.001
	NC vs. SZ-FTD	-3.584*	28	.001
	NC vs. FDR	1.151	28	>.05
	FDR vs. SZ+ FTD	-4.738*	28	.001
	FDR vs. SZ-FTD	2.815*	28	.009
	SZ+ FTD vs. SZ-FTD	1.008	28	>.05

Supplementary Table 11 Independent t-test comparisons of age, education, and WASI IQ for neurotypical controls (NC), first degree relatives (FDR), and participants with (SZ+FTD) and without formal thought disorder (SZ-FTD)

NP types	Model Type	Statistics	p	η^2
Expletives	Kruskal-Wallis	H (3) = 2.659	.447	None
Third-person rigid /total NPs	Kruskal-Wallis	H (3) = 12.510	.006*	.15
2nd person deictic/ total NPs	Kruskal-Wallis	H (3) = 1.877	.598	None
1st person plural/singular/ total NPs	Kruskal-Wallis	H (3) = 6.280	.099	None

Supplementary Table 12 Statistical significance for possible between-group differences in other Noun Phrase types

	Comparisons	U	Z	Sig. (1-tailed)	Effect size (η^2)	Bonferroni correction*
3rd person rigid NPs/ total NPs	NC vs. SZ+ FTD	101.500	-.458	.323	None	>.008
	NC vs. SZ-FTD	112.500	.000	1.000	None	>.008
	NC vs. FDR	58.500	-2.363	.009*	.17	>.008
	FDR vs. SZ+ FTD	23.500	-3.761	.000*	.46	<.008
	FDR vs. SZ-FTD	61.500	-2.231	0.13	.15	>.008
	SZ+ FTD vs. SZ-FTD	91.000	-.895	.185.5	None	>.008

Supplementary Table 13 Pairwise Comparisons of Neurotypical Controls (NC), First Degree Relatives (FDR), Patients with (SZ+FTD), and without Formal Thought Disorder (SZ-FTD) on the use of Third-person Rigid Expressions (i.e., Names) in Mann Whitney U Test
